# Extended spectrum beta-lactamase *Klebsiella pneumoniae* (ESBL-KP) bloodstream infection outbreak in a Neonatal Intensive Care Unit (NICU) in Bangui, Central African Republic (CAR)

**DOI:** 10.1186/s13756-026-01784-x

**Published:** 2026-09-29

**Authors:** Agnese Comelli, Maurice Ebode Ela, Innocent Baluhe Muke, Elisée Danwesse, Siridion Manirarora, Synthia Ningatoloum Nazita, Jean Marie Mafuko, Abdon Isango, Gracia Yasselet, Gael Ndrouma, Bienfait Tombola, Baimi Tao Vaizi, Oluwakemi F Ogundipe, Abdoulaye Sepou, Richard Norbert Ngbalé, Fabiola Gordillo Gomez, Chiara Martino, Anna Farra, Mohamad Khalife, Pilar Garcia-Vello

**Affiliations:** 1 Médecins Sans Frontières - Operational Centre Brussels (MSF-OCB), Brussels, Belgium; 2 Lebanon Branch Office, Middle East Medical Unit, Médecins Sans Frontières, Operational Centre Brussels, Beirut, 1107 Lebanon; 3 Médecins Sans Frontières - Operational Centre Brussels (MSF-OCB), Bangui project, Bangui, Central African Republic; 4 Paediatric Unit, Centre Hospitalier Communautaire Universitaire (CHUC), Bangui, Central African Republic; 5 Gynaecology and Obstetrics Unit, Centre Hospitalier Communautaire Universitaire (CHUC), Bangui, Central African Republic; 6 University of Bangui, Bangui, Central African Republic; 7 Médecins Sans Frontières - Operational Centre Amsterdam (MSF-OCA), London, UK; 8 Luxembourg Operational Research and Epidemiology Support Unit, Medical Department, Operational Centre Brussels, Médecins Sans Frontières, Luxembourg, Luxembourg

**Keywords:** Neonatal sepsis, In-hospital MDRO outbreak, IPC implementation

## Abstract

**Background:**

Multidrug-resistant organism (MDRO) outbreaks in NICUs cause high mortality and strain hospital resources globally, particularly in low-income countries (LICs). In Central African Republic (CAR), limited data on antimicrobial resistance indicate high rates of MDRO. We describe an outbreak of Extended Spectrum Beta-Lactamase-producing *Klebsiella pneumoniae* (ESBL-KP) in a Ministry of Health (MoH) hospital NICU in Bangui, supported by Médecins Sans Frontières (MSF).

**Methods:**

This retrospective case-control study was conducted at the Centre Hospitalier Universitaire Communautaire-MSF project of Bangui. The study included all neonates admitted to the NICU from 25/10/2023, the admission date of the first confirmed case, to 14/01/2024, the discharge date of the last case. Cases were defined as laboratory-confirmed ESBL-KP bloodstream infections (BSI) with similar resistance profiles, differing by no more than three antibiotic classes based on phenotypic testing. Line lists and routinely encoded data were used, and variables were tested to identify significant exposures. Data on outbreak management, infection prevention and control (IPC) interventions and human resources (HR) management were collected.

**Results:**

A total of 242 neonates were admitted during the outbreak period: 44 cases and 153 controls. The incidence rate of ESBL-KP BSI was 29.7 per 1000 patient-days. Mortality among cases was 3.5 times higher (*p* < 0.0001). Twenty-five cases (56.8%) had early sepsis (sepsis suspected < = 3 days of hospitalisation). Case-control analysis identified maternal risk factors for neonatal infection as the most important independent risk factor. The neonatology unit occupancy rate exceeded 100% in early October, and IPC assessments revealed poor adherence to IPC measures at the beginning of the outbreak. A series of IPC interventions in the first month of the outbreak successfully controlled further spread of the infection, and the outbreak was declared over in approximately two months.

**Conclusion:**

The ESBL-KP NICU outbreak reveals gaps in hospital management, with ESBL-KP probably spreading due to high occupancy rate, poor IPC standards, and possibly by multiple simultaneous sources. Access to microbiology and the implementation of IPC practical interventions are critical to identifying and managing outbreaks in LIC.

**Supplementary Information:**

The online version contains supplementary material available at https://doi.org/10.1186/s13756-026-01784-x.

## Background

 Outbreaks in NICUs cause high mortality and strain hospital resources globally, particularly in low-income countries (LIC). Most bacterial pathogens causing NICU outbreaks are gram-negative and sometimes multi-drug-resistant organisms (MDROs) [1]. Among responsible pathogens, the presence and spread of extended-spectrum beta-lactamase (ESBL)- producing Enterobacterales pose a global public health concern [2]. A recent systematic review of the impact of ESBL-producing Enterobacterales in NICUs showed that ESBL *Klebsiella pneumoniae* (ESBL-KP) is the most frequently implicated pathogen in NICU outbreaks, with mortality rates up to 31% in infected neonates [3]. High rates of colonisation, invasive infections, and treatment options for ESBL-positive Enterobacterales have become significant issues in the care of neonates in hospital settings in both high- and low-resource settings [3, 4]. Even if not targeting neonates, it is worth noting that in Bangui, Central African Republic (CAR), a 2021 study reported that 71.2% of children were colonised by ESBL-producing Enterobacterales, indicating community circulation of these pathogens and the potential for transmission to other community members [5]. In a retrospective analysis of neonatal bacteraemia conducted at our hospital, resistance to third-generation cephalosporins was observed in 100% of *K. pneumoniae* isolates tested (2018–2020 data) [6]. Moreover, 85.7% and 77.8% of all Gram-negatives isolated were resistant to first‐line (ampicillin‐gentamicin) and second‐line antibiotics (third-generation cephalosporins), respectively [6]. Since the Doctors without Borders (MSF) sexual and reproductive health (SRH) project opened in Bangui, multiple ESBL-KP outbreaks have been registered in the NICU, notably in 2017 and 2022. The 2017 outbreaks included 25 confirmed ESBL-KP cases, with a 28% case-fatality rate. Antibiotic susceptibility testing showed 100% resistance to ampicillin, gentamicin, cefotaxime and ceftriaxone. Being resuscitated immediately after birth was significantly associated with ESBL-KP infection [7]. The available literature agreed that the most important risk factors for ESBL-KP BSI in neonates are pre-existing maternal colonisation, understaffing, unit overcrowding, suboptimal hygiene standards and current or past (within 30 days) admission of a neonate infected with the same bacteria in the same department [3, 8]. In a published analysis about an ESBL-KP outbreak in the NICU of an MSF project in Haiti, low birth weight and gestational age younger than 36 weeks were identified as risk [9]. In NICUs, mother-to-child transmission during delivery and nosocomial transmission were recognised as the two most common transmission routes of MDRO [8]. However, more recent evidence has not confirmed the association between maternal colonisation and neonatal BSI [10, 11]. The delivery procedures and resuscitation of neonates before their transfer to the NICU are critical moments. Unfortunately, the literature on NICU outbreaks is largely from high and middle-income countries, where diagnostics, determinants, and resources to address outbreaks differ from those in LIC. In the two most recent literature reviews about NICU bacterial outbreaks, one included 39 papers, of which only one was from a middle-income country (India) [8].

In early November 2023, a new cluster of ESBL-KP BSI was identified in the Centre Hospitalier Communautaire Universitaire (CHUC) NICU, and the outbreak persisted until early January 2024. Thoroughly analysing these events is important because doing so helps us gather knowledge that can be used to prevent similar occurrences in the future, address risk factors, and strengthen education, monitoring, and audits related to IPC, especially in projects led by the Ministry of Health (MoH). This study aims to provide crucial information on the factors that lead to outbreaks and interventions that can help block the chain of transmission in low-income settings.

## Methods

### Design and setting

This is a retrospective case-control study conducted at the CHUC MSF OCB project in Bangui, CAR. In CHUC, intensive care for newborns is provided in basic obstetrical and neonatal emergency care (French acronym: SONUB), which is 100% led by MoH and comprehensive emergency obstetric and newborn care (French acronym: SONUC), which is co-managed with MSF. Moreover, the CHUC NICU provides intensive care for newborns born in outpatient health posts partially supported by MSF.

### Study population and definitions

The MSF guidelines for detecting and managing an in-hospital outbreak state that the same resistance profile identified in the same species in at least two patients within a defined space and time defines a probable outbreak. According to this guideline, same resistance profiles mean resistance profiles that differ by no more than three antibiotic classes based on phenotypic testing (i.e., phenotypic results of the antimicrobial susceptibility test: S=susceptible, I=intermediate, R=resistant).

The study included all neonates admitted to the NICU of the CHUC during the ESBL-KP outbreak. The study period lasted from 25th October 2023, when the first confirmed positive case was admitted to the NICU, to 14th January 2024, when the last case was discharged. A case was defined as ESBL-KP BSI. Controls were all other neonates admitted to the NICU, including those with no signs or symptoms of sepsis during their hospital stay and those with bacterial isolates other than KP-ESBL from blood culture. We excluded from the study population neonates with signs of sepsis and negative blood cultures to avoid misclassifying actual cases as controls, given the limited sensitivity of blood cultures in neonates.

### Data collection

In the CHUC NICU, all data on patients admitted to the NICU who underwent blood culture for suspected sepsis are routinely entered into a dedicated linelist (BSI database). This same database was used as the outbreak line list, including all suspected cases in the outbreak period. Case definitions used during the outbreak are reported in Table 1.

**Table 1:** Case definition during the outbreak

SUSPECTED CASES
Neonates (< 28 days) admitted to the NICU with at least one of the following signs or symptoms:
-fever (> 38,0 °C)
- severe hypotonia
- yellow-grey skin colour
- distended abdomen
- brownish gastric aspirates
OR
- contact with a CONFIRMED CASE, defined as being in a neighbouring bed or in the case of twins, even without any symptoms.
CONFIRMED CASES (cases in the case-control analysis)
Cases meeting criteria for suspected cases AND ESBL-KP isolated on blood culture
CONTROLS
Patients without any signs or symptoms of sepsis during the hospital stay
OR
Suspected cases with non-ESBL-KP positive blood cultures

According to the case definition, we decided to include both early- and late-onset neonatal sepsis among cases. Indeed, considering ESBL-KP as an endemic pathogen in the community, as suggested by the existing literature, we wanted to capture all potential sources of infection, from vertical transmission during the delivery from a potentially colonised mother to in-hospital transmission sources.

Data from the BSI database were merged with the routine department database, which collects data on all patients admitted to the neonatology unit, exporting only cases admitted within the same timeframe and not meeting the sepsis suspected case definition (not included in the BSI database). Table S1 shows the variables included in both databases and their definitions.

IPC measures such as hand hygiene (HH), IV catheter bundle (insertion and maintenance), cleaning and disinfection (glow check, using both for training and auditing), and transmission-based precautions were collected from the routine IPC analysis performed by the Stepwise IPC Approach (SIPCA), an MSF-developed tool that aims to collect data that defines the level of progress (a percentage) for IPC within the department. This data was also collected. Moreover, other information on outbreak management, such as staffing and admission management, was collected from the outbreak team’s weekly meeting reports.

### Microbiological investigations

At CHUC, blood culture analysis is outsourced to the SICA laboratory at the MSF France Hospital, which is located in the same city. Blood culture collection and analysis were performed in accordance with international standards.

This laboratory is small and, therefore, limited in the number of samples it can process. For this reason, blood cultures are collected only from neonates who show signs of infection and do not respond to first-line antibiotics. However, during the outbreak, all suspected cases underwent blood cultures.

The microbiology laboratory uses manual incubation, identification is performed by API and antimicrobial susceptibility testing (AST) is carried out using the Kirby-Bauer method in accordance with MSF standard operating procedures based on EUCAST guidelines.

Antibiogo^®^, a digital health application that supports lab techs in delivering high-quality AST [12], is used to read the petri dishes and produce the AST report. Indeed, the application enables laboratory technicians to measure inhibition diameters on AST plates and interpret their results without requiring microbiology expertise.

### Statistical analysis

The data from the linelist were cross-referenced with routine medical data collected and analysed using STATA 17 (College Station, TX, USA).

Variables collected from cases and controls were compared within the same time frame. The odds of exposure among cases and controls were compared using unadjusted logistic regression to estimate odds ratios (ORs) and 95% confidence intervals (95%CI). Adjusted ORs were calculated in a multivariate logistic regression model for variables which resulted significantly associated with the outcome, ESBL-KP BSI, in the unadjusted analysis. Means, medians, ranges, and frequencies were used to describe case and control characteristics. The proportions of strains across different resistance profiles were calculated for bacterial isolates.

## Results

**Fig. 1:**
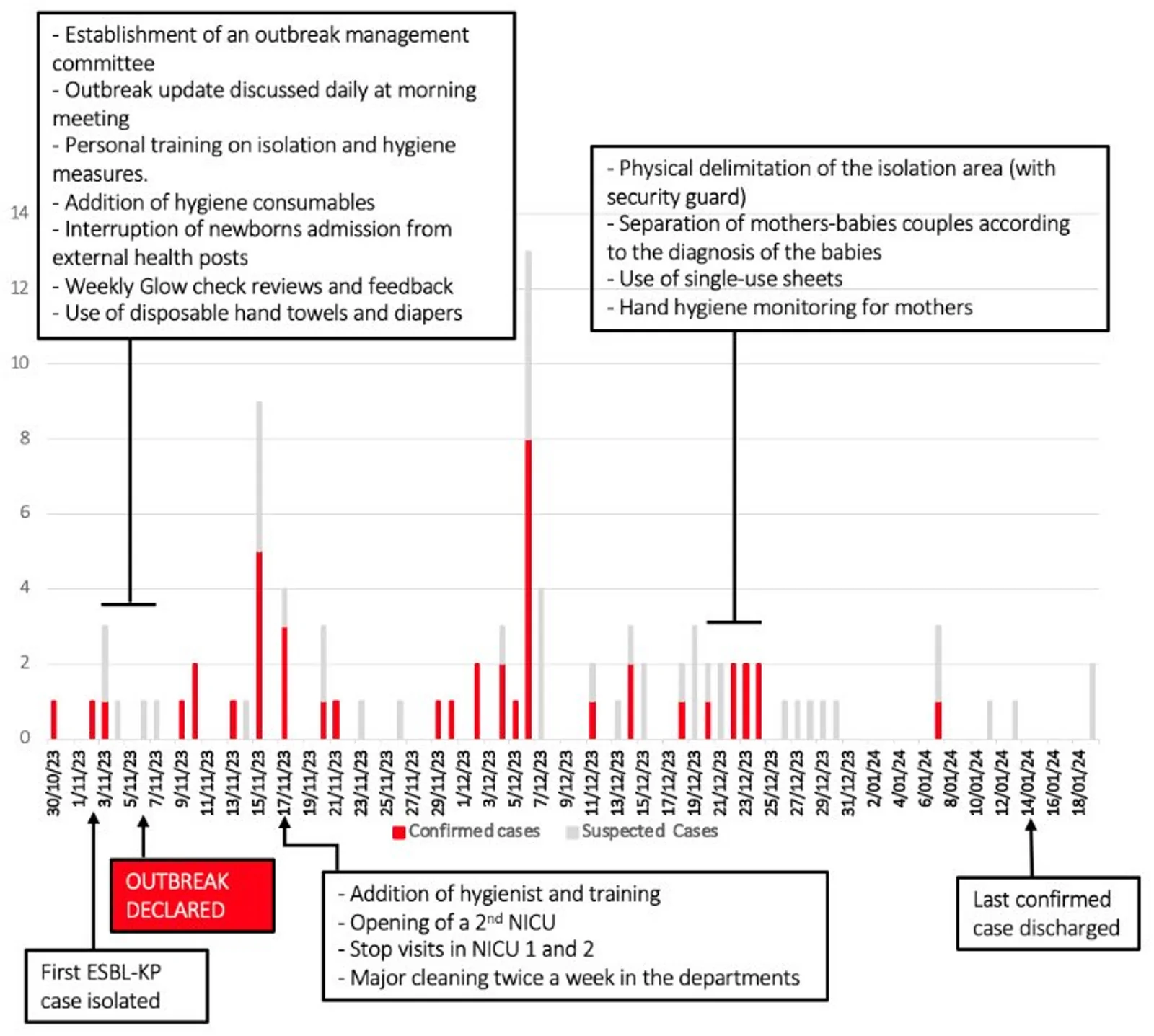
Outbreak curve and intervention timeline

Only four cases of ESBL-KP BSI were recorded at CHUC from January to September 2023. The number of cases has risen since the end of October 2023, and, on November 6th, after the first three cases were identified, a probable outbreak was declared. As shown in Fig. 1, the outbreak committee and the line list were established as soon as the outbreak was declared. The epidemic curve is illustrated in Fig. 1, incorporating the main control interventions. The peak of cases was recorded on 6th December 2023. In the final week of the year, a third block of IPC measures was implemented: physical delimitation of the isolation area with dedicated personnel to ensure compliance, separation of mother-baby pairs by diagnosis, and reinforcement of hygiene practices. Following these interventions, no further cases were reported until the last one on 7th January.

Between the 25th of October 2023, when the first case was admitted, and the 14th of January 2024, when the last case was discharged, 242 neonates were admitted to the CHUC NICU. Baseline characteristics of the overall population are reported in Table 2. Most neonates admitted to the NICU were born in the hospital (83.5%), and the main admission diagnoses were perinatal asphyxia (43.4%) and prematurity/low birth weight (20.3%). One-third received resuscitation at birth.

**Table 2:** General characteristics of patients hospitalised during the outbreak (*n* = 242)

Variable	
Sex, F n(%)	94 (38.8)
Place of birth, n(%)	
Hospital	202 (83.5)
Outpatient - healthcare centre	3 (1.2)
Outpatient – other ^a^	37 (15.3)
Admission diagnosis, n(%)	
Perinatal asphyxia	105 (43.4)
Prematurity/low birth weight	49 (20.3)
Neonatal sepsis	26 (10.7)
Risk of sepsis at birth ^b^	24 (9.9)
Transient tachypnoea	16 (6.6)
Congenital malformation	7 (2.9)
Meconium aspiration	3 (1.2)
Respiratory distress syndrome	4 (1.6)
Neonatal syphilis	1 (0.4)
Other	7 (2.9)
Gestational age, n of weeks (%)	
< 32 weeks	27 (11.2)
32–42 weeks	214 (88.8)
Gestational age, median (IQR)	37 (35–38)
Birth weight, n(%)	
< 1000 g	9 (3.7)
1000–1499 g	21 (8.7)
1500–2499 g	67 (27.7)
≥ 2500 g	145 (59.9)
Birth weight, median gr (IQR)	2750 (1970–3270)
APGAR (1´), n(%)	
>=7	116 (48.3)
< 7	124 (51.7)
APGAR (5´), n(%)	
>=7	166 (69.5)
< 7	73 (30.5)
Resuscitation at birth, n(%)	78 (32.6)
Oxygen received at birth, n(%)	154 (64.2)
HIV positive mother, n(%)	6 (2.6)
Malaria in the mother, n(%)	32 (14.2)
Length of hospitalization (days), mean (sd)	8.6 (10.6)
Time to event ^c^ (days), mean (sd)	7.4 (10.4)
Outcome, n(%)	
Discharged	192 (81.7)
Dead	39 (16.2)
Transferred	5 (2.1)

^a^ home delivery or not declared

^b^ some neonates are kept in hospital observation because one or more criteria for the risk of sepsis are present

^c^ time to event is (1) blood culture collection for cases and (2) death or discharge for non-cases

Ninety-two neonates filled the sepsis-suspected case definition and underwent blood culture collection. From blood culture results, 44 neonates were classified as confirmed cases, 45 had negative blood cultures, and 3 were infected with other pathogens. Controls included the 3 suspected cases with non-ESBL-KP-positive blood cultures and the 150 neonates who never met the criteria for suspected sepsis, resulting in 153 controls. The 45 neonates with negative blood cultures were excluded from the case-control analysis to reduce the risk of misclassification.

The incidence rate of confirmed cases during the outbreak was 29.7 cases per 1000 patient-days, with a proportion of 18.2% (i.e., 44 cases among 242 neonates hospitalised during the outbreak period).

Seventy-eight patients (84.8%) were hospitalised on the same day of their birth; the remaining were readmitted after a median of 4.5 days from birth (IQR 2–17). The median number of days between blood culture collection and patient admission was 3 days (IQR 1–4).

Out of the 44 confirmed cases, 25 (56.8%) were early sepsis (diagnosis of suspected sepsis < = 3 days), and 19 (43.2%) were late sepsis.

Eleven pairs of twins were hospitalised during the outbreak period. In three pairs, both twins were ESBL-KP cases, and in one pair, only one of the two twins had positive blood cultures (the other twin was considered a suspected case, but blood cultures were negative).

Median time to event (case confirmation for cases and death or discharge for cases) was 5.9 (SD 9.10) for cases and 7.8 (SD 10.83) for controls, without a statistically significant difference.

The length of hospitalisation in the case group was, on average, 14.5 days (SD 11.56), and 6.8 days (SD 10.83) in the control group, with a statistically significant difference (*p* < 0.001).

Total mortality among the exposed population was 39 out of 241 (16.2%, one missing data). The mortality rate was 15 out of 44 (34.1%) among cases and 15 out of 153 (9.8%) among controls, with a mean difference of 24.3% (95% CI 9.5% − 39.0%; *p* < 0.0001).

### Case-control study results

In the univariate analysis, several factors were significantly associated with the risk of developing an ESBL-KP BSI (Table 3). These included resuscitation at birth (OR 2.61, 95% CI 1.3–5.3, *p* = 0.006), the presence of a maternal risk factor (*p* < 0.0001), and gestational age at birth of less than 31 weeks (OR 5.3, 95% CI 2–13.9, *p* = 0.0001). Additionally, a significant trend was observed across birth weight categories, with lower birth weights being associated with a higher risk of ESBL-KP BSI (test for trend, *p* = 0.003). Mean time to event was 5.9 days (SD 9.1) in cases and 7 days (SD 10.8) in controls, with no significant difference between groups (*p* = 0.06).

**Table 3:** Case-control analysis results

		Cases = 44		Controls = 153		Unadjusted analysis			Adjusted analysis		
		*n*	%	*n*	%	OR	CI 95%	*P*-value	aOR	CI 95%	*P*-value
Sex	Male	27	61.4	99	64,7	1.15	0.6–2.3	0.68			
	Female	17	38.6	54	35.3						
Place of birth	Outpatient	5	11.4	34	22.2	0.4	0.2–1.2	0.11			
	Hospital	39	88.6	119	77.8						
Resuscitation at birth^a^	Yes	21	48.8	41	26.8	2.61	1.3–5.3	**0.006**	2.5	0.7–8.2	0.14
	No	22	51.1	112	73.2						
Oxygen received at birth^a^	Yes	16	37.2	50	32.6	0.82	0.4–1.7	0.58			
	No	27	62.8	103	67.3						
Malaria in the mother^b^	Yes	7	18.9	15	10.1	2.08	0.8–5.6	0.13			
	No	30	81.1	134	89.9						
Apgar 1’	1–6	23	52.3	81	53.3	0.96	0.5–1.9	0.91			
	7–10	21	47.8	71	46.7						
Apgar 5’ ^a^	1–6	18	41.7	43	28.3	1.83	0.9–3.7	0.091			
	7–10	25	58.1	109	71.7						
Maternal risk factors ^c^	Yes	28	63.6	0	0			**< **0.0001	not estimable *		**< 0.0001**
	No	16	36.4	153	100						
Birth weight ^d^	< 999 g	4	9.1	4	2.6	1	0.2–3.9	**0.003**	0.83	0.3-2.0	0.68
	1000/1499 g	4	9.1	10	6.5	0.1	0.1–0.7				
	1500/2499 g	18	40.9	38	24.8	0.4	0.2–0.6				
	> 2500 g	18	40.9	101	66.0	0.1	0.1–0.2				
Gestational age	0–31	12	27.3	10	6.6	5.3	2-13.9	**0.0001**	7.9	1.4–45.3	**0.02**
	> 31	32	72.7	142	93.4						

^a^ 1 missing data

^b^ 7 missing data

^c^ at least one maternal risk factors among the following: pre-eclampsia, sepsis, meconium-stained amniotic fluid during labour, and premature rupture of membranes

^d^ test for trend

* Due to complete separation (no controls exposed to maternal risk factors), the adjusted OR cannot be reliably estimated. Only the p-value is reported

In the multivariate (adjusted) analysis, the only factors independently associated with ESBL-KP BSI were maternal risk factors and small gestational age (Table 3).

## Microbiology results

The susceptibility profile of the ESBL-KP strains identified is shown in Table 4. The antibiograms confirmed a high level of resistance across multiple antibiotic classes, especially gentamicin (100% resistant) and fluoroquinolones (97.3% ciprofloxacin resistant). Imipenem was the only carbapenem tested; 23 cases (52.3%) showed susceptibility, 20 (45.4%) necessitating increased exposure, and one resistant isolate.

**Table 4:** Sensitivity profiles of the 44 isolated ESBL-KP strains

Amoxicillin/clavulanic acid	Total tested	*N*, %S	*N*, %I	*N*, %*R*
	43	1 (2.3)		42 (97.7)
Cefoxitin	41	3 (7.3)		38 (92.7)
Ceftriaxone/ Cefotaxime	41			41 (100)
Ceftazidime	25		1 (4)	24 (96)
Cefepime	37			37 (100)
Piperacillin/tazobactam	42	19 (45.2)	1 (2.4)	22 (52.4)
Imipenem	44	23 (52.3)	20 (45.4)	1 (2.3)
Aztreonam	6		1 (16.7)	5 (83.3)
Amikacin	44	44 (100)		
Gentamicin	44			**44 (100)**
Tobramycin	44	3 (6.8)	20 (45.4)	21 (47.8)
Ciprofloxacin	37		1 (2.7)	36 (97.3)
Trimethoprim/sulphamethoxazole	32			32 (100)
Colistin	17	15 (88.2)		**2 (11.8)**

Some antibiotics were tested on a case-by-case basis

*S* Sensitive, *I* Susceptible, increased exposure, *R* Resistant

Of the suspected cases, three of 48 patients (6.3%) had microbiological isolation: two *K. oxytoca* and one *Escherichia coli,* all ESBL producers. Blood cultures were negative in the remaining 45 patients.

### Response to the outbreak and IPC results

Figure 1 shows the subsequent main actions taken during the outbreak. The first was to establish an “outbreak committee”. The medical director led this team, which included the IPC team, head paediatricians, and representatives from nursing staff, cleaning personnel, the environmental health team, laboratory staff, the pharmacy, and health promoters. In the first phase of the outbreak, the headquarters advisors supported the team. Weekly formal meetings were held, and daily updates were shared during the healthcare staff’s morning meetings. The infectious diseases and paediatric advisor assisted the local team in clinical management and adapted the empirical and targeted treatment. The IPC advisor collaborated with the local IPC team to establish emergency measures and monitor and adjust them throughout the outbreak management period until the end. It should be noted that MSF supports IPC with specialised human resources, guidelines, monitoring tools, and consumables. A correct IPC-wise setup in the wards and regular educational training are provided in accordance with MSF standards. However, a series of IPC interventions were introduced or strengthened during the reported outbreak. A detailed action plan and focused training on its implementation were provided. Firstly, hand hygiene using alcohol-based hand rub was strengthened to encourage staff and caretakers visiting neonates to disinfect their hands appropriately. Alcohol hand rub (AHR) dispensers were fixed at each baby cot and at the entry of each room. Transmission-based precautions included the mandatory use of gowns upon entering the neonatal service and their removal upon leaving, as well as the introduction of single-use medical equipment for case care. Additionally, disinfection and cleaning protocols for several pieces of equipment used daily were improved. This included regular disinfection of frequently touched surfaces and deep cleaning of all incubators, the rooms and the delivery room. Only mothers were allowed to stay with the neonates; other visitors were forbidden. Admissions to the NICU from external health posts were suspended.

In mid-December, given the ongoing identification of cases, stricter rules were adopted, including direct monitoring of HH among the mothers and security guards to ensure respect for the isolation area. Assessment of specific indicators is conducted monthly. The results during the outbreak and in the 2 months before and after are shown in Table 5.

**Table 5:** IPC monitoring data

	2023					2024	
	Aug	Sept	Oct	Nov	Dec	Jan	Feb
HH %	58	58	65	71	77,5	62	66
Glow check surfaces %	NA	NA	54	58	79,8	78	77
Glow check of non-medical equipment	NA	NA	23,8	46,2	83	76	71
Catheter IV %	63	64	91,1	88,5	90	100	56
Isolation compliance %	100	100	100	100	100	100	100

*Abbreviations*: *HH* Hand hygiene, *IV* Intravenous, *NA* Not available, *SIPCA* Stepwise Infection Prevention and Control Approach

Human resource plans were adjusted to account for staffing needs. The opening of a new NICU for newly admitted neonates and the need for dedicated personnel for isolated patients posed a challenge for identifying qualified staff for neonatal specialised roles during an outbreak. Educational sessions were organised to brief new staff and provide accurate information about outbreak-related interventions and standards.

**Fig. 2:**
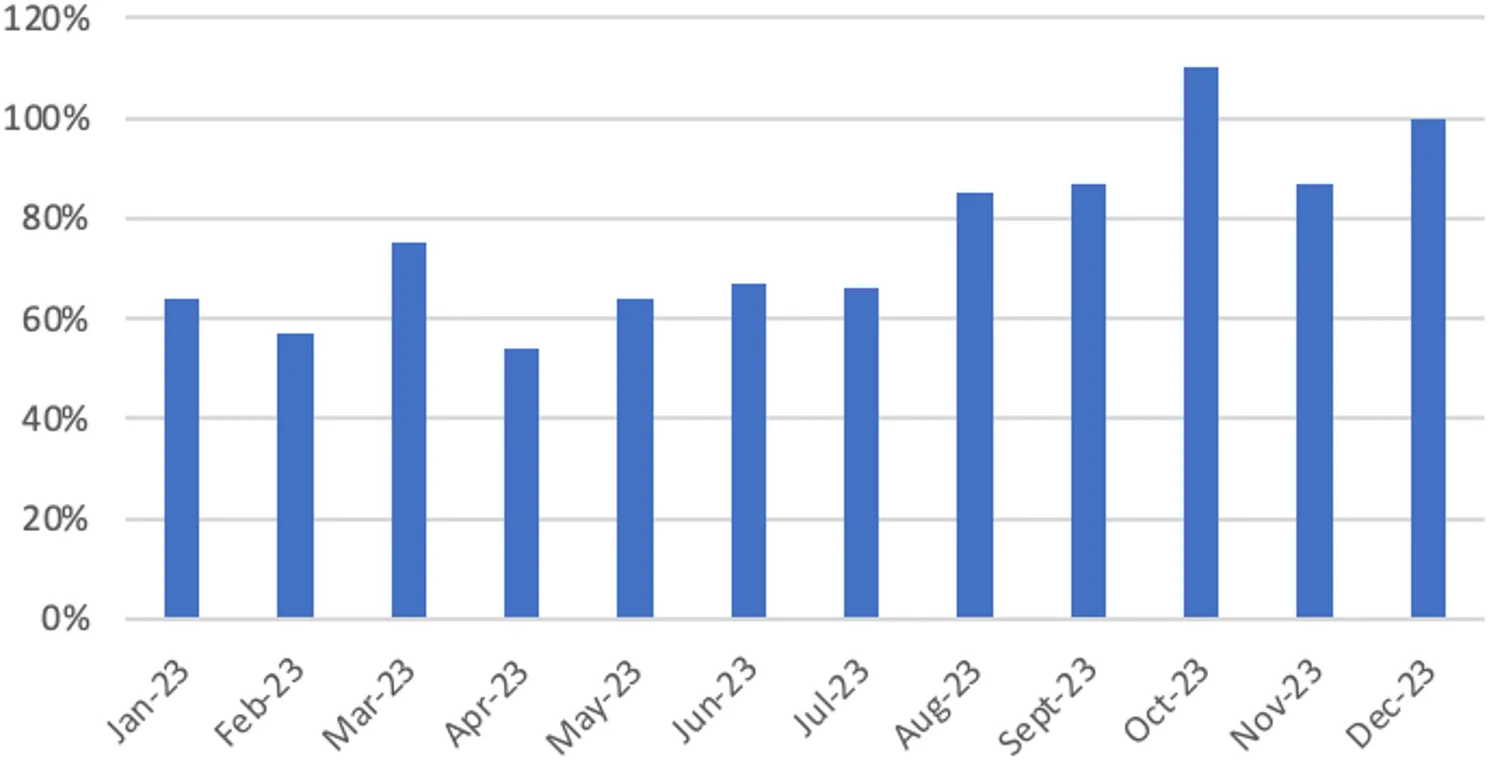
Bed occupancy rate in the neonatology unit (Jan- Dec 2023)

Data from the hospital’s data management reported a high bed occupancy rate in the neonatal department in October, with an upward trend from the beginning of 2024 to the last three months (see Fig. 2). Collaborations were established with other hospitals to transfer non-cases to minimise the exceeding neonatal bed occupancy.

In addition to IPC, the response to the outbreak included optimising clinical management, updating antibiotic treatment protocols, and sustaining epidemiological and microbiological surveillance. In terms of clinical management, neonates fulfilling the suspected case criteria were immediately isolated in a separate isolation room. Confirmed cases were cohorted. Antibiotic treatment regimens were also adapted. First-line antibiotics in neonatal care were ampicillin and gentamicin, as per MSF protocol. During the outbreak, gentamicin was replaced by amikacin as the first-line treatment, in combination with ampicillin, for all suspected cases. This choice was made based on the observed consistent susceptibility in isolated ESBL-KP to amikacin and the goal of reducing meropenem exposure while awaiting blood culture results. When blood culture results for ESBL-KP were formally reported, ampicillin was discontinued, and meropenem was initiated. The overall duration of amikacin administration was 3 to 5 days in combination with meropenem. If the blood culture was negative, the treatment with ampicillin and amikacin was continued.

In the case of a neonate with negative blood cultures deteriorating during ampicillin + amikacin therapy, a multidisciplinary discussion was held to escalate to meropenem empirically.

## Discussion

Circulation of 3rd -generation cephalosporin-resistant Enterobacterales and previous neonatal outbreaks caused by ESBL-KP have been described in LMIC [13], including CAR [5–7, 14, 15]. However, this is the first analysis of a neonatal outbreak formally published in CAR.

During this outbreak, 44 neonates experienced an ESBL-KP BSI. The recorded mortality rate was more than three times higher (34.1% compared to 9.8%), and the duration of hospitalisation was twice as long; both figures show significant differences at the 0.05 level.

The incidence rate of confirmed cases during the outbreak was 29.7 cases per 1000 patient-days. A similar report from an MSF project in Haiti described a reduction in incidence from 50 to 14 cases per 1000 patient-days [9]. The evolution of the incidence rate is unavailable because the outbreak was shorter than the 14-month epidemic experienced in Haiti [9].

Case-control analysis identifies maternal risk factors as the most important independent risk factors. As noted in Table 3, maternal risk factors include the presence of maternal sepsis, meconium-stained amniotic fluid during labour, and premature rupture of membranes. Published data consistently report this risk factor in neonatal MDRO outbreaks [16–21].

We were unable to identify the source of transmission. The sustained circulation of ESBL-producing bacteria in the community, in CAR [5, 15] and in surrounding countries [22–24] supports the assumption that the outbreak could be due to increased hospital transmission of endemic bacteria resulting from poor infection control and hygiene practices.

The hypothesis regarding vertical transmission of ESBL-KP (mother to neonate), along with further horizontal transmission in neonatal services, is supported by the fact that over 50% of cases were diagnosed with early sepsis (diagnosis of suspected sepsis < = 3 days of life), typically resulting from intrapartum transmission from mothers or within the delivery room environment [19, 20]. Unfortunately, we lack data confirming ESBL-producing pathogen colonisation of the mothers. Research has shown conflicting data on the association between maternal colonisation and early-onset neonatal sepsis [10, 11, 16–18]. However, looking more generally to maternal risk factors, a meta-analysis demonstrated a high prevalence of early-onset neonatal infections among newborns whose mothers had infections or risk factors, aligning with our findings [21]. On the other hand, although the ESBL-KP strains appear to differ slightly phenotypically, we cannot rule out clonality due to the limitations of disk diffusion methods and the absence of molecular testing. Horizontal transmission in neonatal services is readily explained by the high and rising bed occupancy rate and poor IPC indicators prior to the outbreak, as shown in Fig. 2; Table 5.

Finally, horizontal transmission of ESBL-carrying genes cannot be excluded.

As mentioned before, no neonatal risk factors have been identified, in contrast to similar studies, in which low birth weight, resuscitation, and low 5’ and 10’ Apgar scores at birth were significantly associated with case status [9]. If high-rate maternal colonisation was confirmed, prospective cohort studies could clarify whether variables such as birth manipulation and prematurity represent significant risk factors for invasive bacterial infection in cases of maternal colonisation.

MDRO outbreak management in low-resource settings is challenging, mainly due to the lack of a microbiology laboratory to confirm the outbreak, human resource shortages, overcrowding of paediatric care facilities, shortages of consumables, and challenges related to IPC measures implementation. Proper and consistently implemented IPC activities are the most effective way to prevent the onset of an outbreak, mitigate escalation and control it. Unfortunately, in most LICs, IPC activities are still not consistently and adequately included in basic healthcare practice, and investment in professional curricula remains limited in most healthcare facilities [25].

CHUC hospital’s co-management arrangement with MSF has enabled access to a quality-assured microbiology laboratory. In settings without a microbiology laboratory, periodic or exceptional spikes in neonatal mortality may, in fact, be due to outbreaks of bacteria resistant to first-line treatment. Access to microbiology enables prompt identification of the outbreak, initiation of prevention activities, and adaptation of antibiotic treatment.

Unfortunately, treatment access is another critical factor in outbreaks caused by MDROs.

On one hand, access to carbapenems seems essential for providing a chance of survival from an ESBL-producing Enterobacterales invasive infection. On the other hand, the absence of a robust regulatory system for drug circulation in LICs increases the likelihood of antibiotic misuse [26]. This is particularly worrying given that, despite limited access to carbapenems, isolating carbapenem-resistant pathogens is not uncommon [27].

Another advantage of access to the microbiology laboratory is that cumulative results may help in reviewing and adapting antibiotic treatment protocols. The 100% resistance of isolated strains to gentamicin, a first-line treatment for sepsis in newborns at MSF and prophylaxis for neonates born to mothers with diagnosed systemic infection, is concerning. We need to analyse additional results and integrate them with patient outcomes to assess the impact of this phenomenon and decide whether treatment guidelines should be adapted.

Compared to access to bacterial cultures and carbapenems, basic IPC measures are less expensive and easier to implement, especially in low-resource settings. By contrast, a large survey of clinicians in Sub-Saharan countries reports that access to IPC remains limited [28]. As shown in this outbreak description, one of the challenges was the recruitment of qualified staff, a pillar for the respect of IPC standards and recognised as a risk factor for the onset of neonatal outbreaks [3].

Although, for many IPC measures, a clear analysis of the impact of individual IPC interventions is lacking, we would like to report the 10 priority IPC interventions we implemented after context-specific adaptation of existing MSF IPC guidance. Unfortunately, we could not establish a clear link between any specific intervention and the outbreak’s resolution. Although the last interventions shown in Fig. 1 appear to have reduced the number of cases, the earlier interventions took time to implement, and we cannot rule out their contribution to the outbreak’s eventual control Table 6.

**Table 6:** 10 priority IPC interventions implemented after adaptation from existing guidance

IPC intervention
Water free care
Peripheral IV access bundle
Early isolation and providing personal kit for suspected cases until microbiological results
Dedicated staff and equipment
IPC leadership in surveillance and audit
Rationalise activities and staff movements in NICU
Create a separate room for new admission in the NICU
Hand Hygiene strengthening
Bed reduction to increase the space between beds
Terminal cleaning after each patient discharge

This study has several limitations. First, the lack of data on maternal colonisation and molecular typing could have reduced our capacity to understand the true dynamics of outbreaks. Control of confounders is inadequate in the case-control analysis, as matching by age was not possible because age data for controls were unavailable from the routine department database. We found that matching solely by sex was not significant in this study. Other potential transmission routes were not investigated, such as incubator, contamination of maternal milk during handling and storage or infusion bags. Moreover, the delivery mode was missing for most of the controls because it is not included in the routine department database.

Finally, the choice of including early-onset neonatal sepsis (EOS) among cases might be controversial. However, when an endemic pathogen is driving an outbreak, including EOS in the population helps highlight potential prevention strategies to protect neonates from “community exposure”. In practice, this means strengthening protective measures during delivery and early neonatal care in both hospital and non-hospital settings. To ensure the paper’s relevance to similar contexts in LMICs and to endemic pathogens, we believe this approach could be more informative.

## Conclusion

AMR poses a significant threat to vulnerable populations such as neonates [29], even greater where access to microbiology and appropriate antibiotic treatment is limited. In addition, prioritising IPC measures could be a feasible first step to reduce in-hospital MDRO outbreaks. More broadly, a structured approach is needed to slow AMR in LIC, in line with WHO recommendations to establish a National Plan against AMR that must address awareness, prevention, care, access, and their sustainability simultaneously [30].

## Supplementary Information

Supplementary Material 1.

## Data Availability

The data that support the findings of this study are available on request from the corresponding author.
