# Supplementary material for: Extended spectrum beta-lactamase Klebsiella pneumoniae (ESBL-KP) bloodstream infection outbreak in a Neonatal Intensive Care Unit (NICU) in Bangui, Central African Republic (CAR)
Source: Antimicrob Resist Infect Control. 2026 Sep 29;15:121. doi: 10.1186/s13756-026-01784-x (PMC13621614; doi:10.1186/s13756-026-01784-x)
Supplement: Supplementary file 1 [file 13756_2026_1784_MOESM1_ESM.pdf]

Table S1. BSI database and routine department database variables

VARIABLE	CHOICE/FORMAT	OUTBREAK LINE LIST	Routine NICU department DB
sex	F/M	X	X
place of birth	CHUC/health posts	X	X
birth date	[date]	X	
mother gestational age	[weeks]	X	X
birth weight	[grams]	X	X
Apgar scores at 1 and 5 minutes	[number]	X	X
delivery mode	vaginal / caesarean section / ventouse	X	
history of mother antimicrobial use	Yes/No	X	X
administration of resuscitative procedures at birth	Yes/No	X	X
duration of resuscitative procedures	[minutes]	X	X
administration of oxygen at birth	Yes/No	X	X
use of Ambu at birth	Yes/No	X	
cardiac massage performed at birth	Yes/No	X	
admission date in NICU	[date]	X	X
admission diagnostic	Neonatal asphyxia / meconium aspiration / congenital anomaly / prematurity-LBW / sepsis risk factors / sepsis / respiratory distress of the neonate / neonatal syphilis / transitory tachypnoea of the neonate	X	X
maternal risk factors	pre-eclampsia / sepsis / meconium-stained amniotic fluid during / labour / premature rupture of membranes	X	X
maternal VIH	Yes/No	X	X
maternal syphilis	Yes/No	X	
maternal malaria	Yes/No	X	X
onset of signs and symptoms of sepsis	[date]	X	
type of signs and symptoms of sepsis	Fever / jaundice / abdominal distention / skin rash	X	
outcome	Death / Cured / Transferred	X	X
discharge date	[date]	X	X
blood culture collection date	[date]	X	
results of microbiological and susceptibility testing	Isolate and AST	X	
antibiotic treatment received	ATB list	X	
